# A household perspective on access to health care in the context of HIV and disability: a qualitative case study from Malawi

**DOI:** 10.1186/s12914-016-0087-x

**Published:** 2016-04-01

**Authors:** Stine Hellum Braathen, Lifah Sanudi, Leslie Swartz, Thomas Jürgens, Hastings T Banda, Arne Henning Eide

**Affiliations:** Department of Health, SINTEF Technology and Society, PB 124 Blindern, 0314 Oslo, Norway; REACH Trust, P.O. Box 1597, Lilongwe, Malawi; Department of Psychology, Stellenbosch University, Private Bag X1, Matieland, 7602 South Africa; Department of Psychology, Stellenbosch University, Alan J Flisher Centre for Public Mental Health, Private Bag X1, Matieland, 7602 South Africa; LHL International Tuberculosis Foundation, Grensen 3 (7th floor), 0159 Oslo, Norway

**Keywords:** Disability, HIV, Care, Household, Malawi

## Abstract

**Background:**

Equitable access to health care is a challenge in many low-income countries. The most vulnerable segments of any population face increased challenges, as their vulnerability amplifies problems of the general population. This implies a heavy burden on informal care-givers in their immediate and extended households. However, research falls short of explaining the particular challenges experienced by these individuals and households. To build an evidence base from the ground, we present a single case study to explore and understand the individual experience, to honour what is distinctive about the story, but also to use the individual story to raise questions about the larger context.

**Methods:**

We use a single qualitative case study approach to provide an in-depth, contextual and household perspective on barriers, facilitators, and consequences of care provided to persons with disability and HIV.

**Results:**

The results from this study emphasise the burden that caring for an HIV positive and disabled family member places on an already impoverished household, and the need for support, not just for the HIV positive and disabled person, but for the entire household.

**Conclusions:**

Disability and HIV do not only affect the individual, but the whole household, immediate and extended. It is crucial to consider the interconnectedness of the challenges faced by an individual and a household. Issues of health (physical and mental), disability, employment, education, infrastructure (transport/terrain) and poverty are all related and interconnected, and should be addressed as a whole in order to secure equity in health.

## Introduction

The right to health care for all is protected in several international conventions and declarations, including the Universal Declaration of Human Rights [1], the Convention on the Rights of Persons with Disabilities [2] and the Declaration of Alma Ata [3]. Despite these, a vast majority of the population in low-income countries face problems with many aspects of access to health care, including affordability, availability, accessibility, acceptability and appropriateness [4–6]. It is well established that the most vulnerable segments of these populations, such as people with disabilities, face increased challenges in accessing health care, as their vulnerability amplifies problems of the general population, which again implies a heavy burden on their main caregivers as well as immediate and extended households [7–17]. However, research falls short of explaining the particular challenges experienced by these individuals and households. As in other areas, to build an evidence base on access to health care from the ground, a nuanced case study is well suited to the exploration and understanding of individual stories [18, 19]. Furthermore, in the field of disability studies, the importance of single case narratives has been highlighted as central to the understanding of the experience of disability [20]. It is important to be able to listen to the individual experience and to honour what is distinctive about the story, but also to use the individual story to raise questions about the larger context [20]. In the context of a larger study, in this particular area we are using a single case study in order to explore and analyse the roles that culture and context play in shaping illness experiences and behaviour [21]. This article aims to explore the particular challenges of vulnerable groups in accessing health services through an in-depth exploration of one household in the context of HIV and disability in rural Malawi.

## Background

In this article disability is conceptualised through a bio-psycho-social approach, emphasising the interactive and dynamic nature of disability; acknowledging both individual health status as well as personal, environmental and cultural factors in the disability experience. Disability is commonly divided into categories of physical, mental, intellectual and sensory (visual/hearing). The approach is seen as a contributor to equal rights, opportunities and participation in society for people with disabilities [14, 22–24]. It is estimated that people with disabilities make up approximately 10–20 % of the world’s population, of whom 80 % live in low-income countries [14, 25, 26]. A growing body of evidence describes people with disabilities as the poorest, most marginalised and vulnerable segment of any population [13–17, 27–29]. People with disabilities often have complex health needs related to their disabilities, in addition to the same health needs as everyone else [26]. Despite these, a vast majority of individuals with disability living in low-income countries have limited access to health and social services [12, 14, 25, 26]. As a result, they are at risk not only for poor health but also for not realising their full potential and participation in society, and thus not realising their human rights [30–32].

The consequences of the vulnerability and exclusion experienced by people with disabilities extend beyond the individual. Research from southern Africa shows that households with disabled family members are often worse off on indicators of poverty and living conditions compared to households without disabled family members [7]. In Malawi, one of the poorest countries in the world [33], people with disabilities have lower school attendance, employment, income and access to health and social services compared to their non-disabled peers [12, 34]. Similar patterns are found at household level, comparing households with and households without disabled family members in Malawi [34].

For more than three decades, the HIV and AIDS epidemic has placed enormous burdens on individuals, families, communities and countries [35]. Households and families, especially in low-income contexts such as those in sub-Saharan Africa, are affected in myriad ways [36–39]. It is only recently, however, that disability and HIV/AIDS, and the complex relationship between the two, have featured on the radar of the international research community (See for instance: [40–46]). On the one hand, the side effects of the virus and medication can result in disabling conditions. On the other hand, people with disabilities are at equal or increased risk of being infected with HIV compared to non-disabled people [42, 43, 45]. Research has however shown that people with disabilities face a range of barriers in accessing sexual health education and care, ranging from practical (for example, information unavailable in Braille/sign-language, physical access to facilities) to attitudinal (health care workers may believe that people with disabilities are asexual, and thus not in need of sexual and reproductive health services) [42–45].

The concept of care is highly contextual and difficult to define. In the broadest sense it encompasses all practices which aim to maintain the world by meeting the needs of ourselves, of others and of our environment [47–49]. It is a human practice which is essential for the survival of individuals and societies [50]. Central to this paper is the ethics of care, a relational theory which sees human beings not as isolated entities, but as dependent and interdependent beings [51]. Care happens at all levels of society, for example, in the household, extended households, communities, institutions, and governments [52]. Provision of care is dependent on the social, relational, economic and political context in which it takes place. As such, it is highly contextual, influenced by peoples’ way of life, their culture and their values [52]. In many African cultures individuals are not seen as belonging to themselves or their families, but to society as a whole. Due to the reciprocal nature of the family and community, individual welfare impacts on the larger community. From such a perspective it is important to consider the whole community, rather than its individual members alone [53].

In a sub-Saharan African context, with under-resourced and underdeveloped health services, health policies and health institutions often endorse the family as the dominant care provider for chronic illnesses. As a result, the majority of the care burden is placed on the immediate or extended family, most often the women [8, 9]. Family and community care has great societal benefits, potentially saving professional and economic resources, but it may place a burden on the informal, unpaid caregivers [54]. Family caregivers play a crucial role in the seeking and giving of health care for both adults and children. Primary caregivers are usually members of the same household or extended household as the immediate care seeker, and hence illness and care seeking becomes a household issue, more than just an individual issue [55]. Caregiving responsibilities often place added burdens on already impoverished households. Burdens are financial, such as transport costs, health care fees, loss in household productive activities and livelihood resources, and emotional and stress related [10, 11, 54, 56–60]. Studies from across the world found that the relative financial burdens were particularly high in low-income countries [54]. The competence and ability of the caregiver, especially in sub-Saharan Africa, may be compromised by poverty, insufficient material resources and the need to sustain livelihood activities [8, 9].

Children and adults with disabilities are often more dependent on care throughout their lives than are their non-disabled peers [10]. The double burden of caring for a disabled family member, in addition to caring for the household, has been referred to as ‘double-trouble caring’ [11]. Added to this, HIV and AIDS also place enormous care burdens on families and communities [35]. The importance of support for both the caregivers and the care receivers is crucial to explore and understand, in order to relieve caregiver burdens and ensure the best possible care for the care receivers [54]. It has been argued that if caregivers receive adequate support, the burden of caregiving can be lessened, and their ability to give care can be enhanced. Support needs of caregivers include financial (free health care, cash transfers), emotional (social networks, counselling), practical, training, respite care and dealing with stigma, discrimination and social exclusion [9, 11, 56, 57, 59]. In addition to support from the health services, caregivers also need community and environmental support [10].

There is a lack of empirical evidence on caregiver burdens and need for support from poor contexts in general, and Malawi in particular [11]. It is important to begin developing a nuanced picture of care in these contexts. Structures of care and support, both for the care receivers and the caregivers, must be based on real needs best identified through in-depth, cultural, contextual and individual evidence. Care is patterned contextually and individually, and both the challenges and the benefits of care may be experienced differently in different settings and by different people [10, 61]. We will use a single case study approach to provide an in-depth, contextual and household perspective on barriers, facilitators, and consequences of care provided to persons with disability and HIV.

## Methods

The case study presented in this article forms part of a larger, qualitative study exploring access to HIV and AIDS services for people with disabilities in Machinga District in Southern Malawi. Data collection was carried out in February 2014. We utilised a case study approach, which is useful for in-depth investigations of contemporary phenomena in real-world contexts [62]. The case study presented in this article was chosen to represent the perspective of a household comprising a member with both disability and HIV. The case provides an in-depth exploration of a person from the perspective of the individual, her primary caregiver, and health service providers at the health services they utilise. This gave us an opportunity to gain multiple perspectives on the same phenomenon and the same individual [62–64]. The case was identified through a community based rehabilitation worker engaged by Malawi Council of the Handicapped (MACOHA) in Machinga. MACOHA is a government organisation providing rehabilitation programmes and services towards the empowerment and inclusion of people with disabilities in Malawi. The case presented in this article exemplifies and explores experiences and implications of care from the perspective of a vulnerable, poor and rural household. To provide a contextual framework for this case we use information from interviews with health providers from relevant health services in the study area. These include one nurse, one HIV Testing and Counselling (HTC) provider, two Antiretroviral Therapy (ART) providers and two expert clients. Except for the nurses, none of the health providers were professional health workers. The HTC and ART providers had received some basic training through the health system, while the expert clients were HIV positive patients from the local community volunteering as lay-counsellors. All the health providers were residents of the local community.

We applied two major data collection techniques: in-depth, individual interviews with the relevant informants and direct observations through visits to their home, local community and health facilities. The interview themes included socio-demographic/economic information about the individual case and the household, disabilities, living with HIV and AIDS, health seeking, family support and other support. Themes for health providers included information about HIV and AIDS services, particular services for people with disabilities, and knowledge on and experience with people with disabilities. The interviews were recorded, transcribed verbatim and translated into English. The interviews were carried out in the preferred language of the participants, and some interviews were carried out by an English-speaking researcher together with a local language interpreter.

The overall goal of case studies is to provide thick descriptions that enable us to get a better understanding of the study phenomenon. Case studies are particularly helpful in providing information about phenomena which are rare or about which little is known in the literature, as is the case with the current topic – HIV and disability in Malawi. Insights drawn from single case studies are not immediately generalisable but provide rich information which can form the basis for further study and research [62, 63]. In the data analysis we started by highlighting the most important themes emerging from the case. We then matched the patterns found in the case with patterns found in theory [62, 63].

People with disabilities potentially have increased vulnerability to exploitation and abuse [13–17]. This is also true regarding their involvement as research participants [65]. The importance of involving vulnerable populations in research has been advocated for by politicians, researchers and user groups, but it is important for researchers to recognize the potential challenges of this involvement [65–68]. At the heart of any research project involving human informants is informed consent. For some people with disabilities, the nature of their disability may compromise their ability to fully understand the information given to them, and thus give informed consent. It is the responsibility of the researchers to ensure that the informants meet the criteria for this [66]. For informants who do not meet the criteria, consent can be obtained from a guardian, and assent from the primary informant. Alternatively, a proxy respondent can be used in place of the primary informant [66]. The study followed strict ethical principles for research. This includes ensuring that all participation was voluntary and consensual, with no negative consequences anticipated from participation. Furthermore, anonymity of the informants is ensured in any presentation of the data, and permission to use the data in publications was obtained from all informants. Ethical approval was obtained from the National Health Sciences Research Committee (NHSRC) of the Ministry of Health in Malawi (Reference number “Protocol #: 1051”).

## Results

### The study context

Malawi is one of the poorest countries in the world, with a population of approximately 16 million, and an estimated two thirds living in poverty [33]. About 85 % of the population live in rural areas, where the highest poverty levels and poorest health outcomes are found [69]. Machinga district is located in Southern Malawi. Machinga District Hospital is a secondary health facility located in Liwonde, a semi-urban town. The rest of the district is rural, bordering Liwonde National Park, Lake Chilwa, Lake Chiuta, Shire River and the international border with Mozambique to the east. The district covers an area of 3771 square kms, and has a population of 369,614 people. The hospital is a referral facility to a number of rural primary health care clinics and health centres. All the health facilities in the district are faced with a general shortage of medical personnel, supplies and treatment [70].

### Procedures for HIV testing, counselling and treatment in Machinga

HIV Testing and Counselling (HTC) and Antiretroviral Therapy (ART) in the district are carried out at primary health care facilities. Despite the overall shortage of personnel, equipment and medication at health facilities in the district, HIV services are relatively well equipped and staffed. Health personnel at these facilities, however, told us that due to lack of transport at the facilities they were prevented from carrying out much needed HIV outreach services and from following up patients in the communities.

When patients test positive for HIV they are registered and counselled on the implications of being HIV positive and living with the virus. Next, patients are referred to a clinician at the same facility, usually the following day. The clinician evaluates if ART is required. If it is, patients are initiated on ART and receive counselling on when and how to take the medication, special dietary recommendations while taking the medication and possible side-effects. During this counselling the patients are encouraged to bring a guardian, so that the patient and guardian can learn together how the drugs should be taken, and the dangers of defaulting.

During the first six months on ART the patients collect their medication monthly. When they have followed the treatment successfully over a six-month period they get medication for three consecutive months at a time.

### Procedures for patients with disabilit**i**es at the facilities

All the ART providers we spoke to said that they ensure that they see people with disabilities first, so that these patients can leave the clinic in good time. This is common procedure at most health facilities in Malawi, as explained by an expert client:

*When these people* (patients) *come here they all pile the health passport books there, and if we see that there is one with disabilities in the group we usually ask the group if we can first treat the disabled one, and in most cases the majority accept our plea because we want them to go and reach home in good time because they have difficulties in walking.*

At the health facilities providers sometimes face problems providing appropriate care for people with disabilities. This is mostly due to communication problems when the patients have visual, hearing or speech impairments. An ART counsellor explained to us how these challenges were solved:

*… what we do is we call the guardian who usually communicated with him at home, so that he can be communicating with him whatever we are telling him in the counselling session. The guardians are there helping me to hear what the clients were trying to tell me, and without them nothing would have been done.*

Overall, the health providers felt that health services were accessible to people with disabilities. One expert client said the following on the differences in access for people with and people without disabilities:

*No, there is no difference when accessing treatment because when these disabled people are coming here they are escorted by those who are normal. That means that they are cared for starting from their communities up to here and they are not stigmatized in any way.*

### Case study

The case study presents the story of a household experiencing disability and HIV in a poor, rural Malawian village. The story has been altered slightly and names have been changed. This was done to secure the anonymity of the informants. The pseudonym of the woman this case story is based on is Naphiri. She is 33 years old, living with HIV and paralysis in her right hand and leg. We interviewed Naphiri in person, her mother and health providers at the health facility where she received health care. Naphiri’s mother does not know her own age. She is married to Naphiri’s father, and together they have 15 children, of whom seven are still alive. Naphiri lives in her parents’ house together with her parents, her own 11-year-old son, her divorced sister, and the sister’s two children. In addition to the sister, two siblings live in the village, two brothers in South Africa and one brother in Blantyre (a city in Malawi). Naphiri’s household makes a living from self-subsistence farming, a small business baking and selling banana fritters, and financial support from the brothers working in South Africa and Blantyre. Naphiri has two sons, but her oldest son (13 years) lives in Blantyre with her brother. The father of Naphiri’s sons is not involved and provides no financial or practical assistance.

Naphiri became disabled four years ago. She explained that she fell sick, was unable to speak and had sores all over her body, and during the illness she became paralysed in her right hand and leg. She had gone to the hospital where she was tested positive for HIV and started on ART. She explained that she tested positive for HIV around the same time that she became disabled. Naphiri’s mother, on the other hand, told us that Naphiri became paralysed a few months after she was diagnosed with HIV and had started on ART, and thus suspected that the medication caused the paralysis. Of the initial illness, the mother said that Naphiri had been suffering from headaches and body pain, and that she had been *‘speaking nonsense’*, and therefore they had thought that *‘she was bewitched or was attacked by evil spirits’,* because they did not know what was happening until they were told that she was HIV positive.

The mother said the following about the time Naphiri fell ill:

*Naphiri was living in (Northern Malawi), she was working there. We only received a phone call through her friends that she is ill and her father immediately went to see her and she was found in a very critical condition. She was very ill and her father hired a vehicle that took her to the health facility where she was admitted and I was called to take care of her as a guardian. And two days later I was called by a health worker who confided in me that my daughter Naphiri is HIV positive and I was advised to attend the counselling session on how she would be taking the drugs.… We stayed in the health facility for three weeks and then we were discharged.*

The mother seemed to know more than Naphiri herself about what had happened before, during and after she received health care. Naphiri told us that she had not been asked at the hospital if they could take an HIV test, but her mother told us that *she* had been asked for permission for them to test Naphiri. Note that Naphiri was very sick at the time, and this may be why they asked for permission from the mother instead of Naphiri herself. The clinician had also shared the results from the test with the mother before sharing it with Naphiri.

Naphiri’s family knows that she is HIV positive, but they have not told anyone in the community. The mother explained why and who decided they should not disclose to the community:

*It was my eldest son who is staying in Blantyre (who decided we should not disclose). It was because the issue will bring shame on us, as people might be speculating a lot of things because of this issue.… If they are not the family members they do laugh and gossip because they are not our relative. But if you are related to her we feel sorry and we have accepted that God chose her to be like that and it is our responsibility to look after her.*

Naphiri goes to the health facility every three months to collect her ART. It takes her three hours each way to walk to the nearest facility that provides the drugs. Including all the walking and the waiting at the facility, she is usually gone for the whole day. Sometimes the family hire a bike for her, but the cost of this is very high. She normally goes alone, but in the beginning her mother had to assist her, or she collected Naphiri’s medication for her at the health facility. Her mother told us that:

*… because she was unable to walk I was going to the health facility to collect her drugs, and when she started walking I took her to the health facility and introduced her to the health workers and she go there for herself, and what we do is to hire for her a bicycle so that she can move fast. (She uses a bicycle)… because we feel sorry for her.*

The mother talked about the challenges she faced when she was escorting Naphiri to the facility:

*There was none to cook for the family, and I was waking early in the morning cooking nsima* (maize porridge – the staple diet in Malawi) *and put it in the food warmer so when one is hungry the food was already there for them. But it was really problematic.*

The mother talked about how the household cope financially:

*I do bake some fritters and sell them and if she wants to go there (health facility) I call for the bicycle to take her from here to the facility and back home. The other source of money is our children who live in other parts of the country. They assist us by sending us money that is also used to help Naphiri. But if there is no money she walks from here to the facility and back home.*

Since she became disabled Naphiri has struggled to do the things she did before. She can still use one hand to wash her clothes, farm, sweep and bath herself, but she cannot cook, and she cannot work as a security guard as she did before she became disabled. She works in the garden, although she struggles with only one functioning arm. Naphiri said that she wishes she could be whole again, and build her own house. Her mother said that the main problem with Naphiri’s disability is that she can no longer help with household chores the way she could before she became disabled. Naphiri said:

*I am always assisted by people.*

## Discussion

It has been extensively documented that disability places limitations on many aspects of participation, and thus also on access to health and other services. In a context where most people are poor and experience challenges accessing health care, the situation for people with disabilities is even more difficult [13–15, 17, 26, 29]. The result is that people with disabilities are among those with the poorest access to health services [14, 25, 26]. The probability of exclusion increases with level of activity limitation, or severity of disability [12]. Implications of these challenges and exclusion extend far beyond the individual, and also impact on families, both the immediate and extended households [7, 10, 12]. To explore and understand the particular challenges experienced by these individuals and households in accessing health care, this paper has presented the single case study of Naphiri. This case study allows us to explore the roles of culture and context in shaping illness experiences and behaviour. While we recognise that we cannot address all issues arising with this limited data, the case study raises a number of issues that are crucial to consider for the larger context.

Naphiri is dependent on assistance and support in a number of aspects of accessing and utilising health care. She sometimes needs support getting to and from the health facility for testing, treatment and to collect her medication. Furthermore, at facility level when she was first hospitalised, a guardian had to be present with her to communicate and make decisions on her behalf. Also, at facility level, many health services are organised in such a way that they are dependent on patients having support from a guardian, family member, friend, or others. Finally, decisions related to seeking or utilising health care, or disclosing health status, are made at household or extended household level. Naphiri and her family live in a typical communal African culture, focussing on the interdependence of the household or community members, rather than on the independence of individuals [53]. In addition to the interdependence of the members of the immediate household, the family are also supported financially, emotionally and practically by family members residing outside the immediate household (the extended household). As a result, the implications of Naphiri’s disability and illness extend far beyond her as an individual, affecting the entire household, immediate and extended. The family face many problems related to Naphiri’s illness and consequent quest for health care. Many of these problems will be the same for her non-disabled peers in the community. The consequences for Naphiri and her family, however, are amplified because they have to deal with the consequences of her disability in addition to her being HIV positive.

The practical challenges faced by Naphiri and her household are typical of those found in the literature [54, 59, 60]. In Naphiri’s immediate and extended household the women largely carry out the day-to-day practical care duties, while the men contribute financially. The overall care choices, however, are made by the men, such as the father being the one to collect her at the onset of her illness and take her to the hospital. Once the decision to seek help from the hospital was made, the mother was called to stay with her and care for her at the hospital. Adding to this, the decision not to disclose Naphiri’s HIV status to people outside the family was made by her eldest brother, and he and another brother are also responsible for providing financial assistance to Naphiri and the rest of the household. In a household like Naphiri’s, the daily tasks are carefully distributed among the family members, and each member is crucial for the wellbeing and survival of the overall family. When one family member, like Naphiri, becomes unable to carry out her chores because of her disability, this places an added burden on all the other household members who have to do her chores for her. Adding to this Naphiri is HIV positive, which means that she has to take medication and visit health care facilities on a regular basis for the rest of her life. Sometimes she can go to the facility on her own, but at other times she needs a family member to assist her. Naphiri’s mother explained the practical difficulties she faced when she could not carry out her regular chores because she had to assist Naphiri.

At health facility level, general HIV services are structured in a manner which requires support from a guardian at certain points of the HIV counselling and treatment pathway. For instance, health services assume that people with HIV will have family members or others to assist with adherence, knowledge about the condition and patient, psychosocial support, dietary requirements, prevention and appointments as necessary. Guardian support is for most households synonymous with household or family support [55], as it is in the case of Naphiri. Treatment adherence is strengthened with this support; it is of inestimable importance to the patient and to the health service, but it comes at a cost to the guardians, and sometimes to the patients themselves. Naphiri’s case reveals her need for support getting to and from the health facility. For people with visual or hearing impairment many experience additional problems, such as navigating within the facility and communication [71]. Interviews with health workers illustrate that the providers themselves do not experience these as big problems, as the families of people with such impairments provide the necessary support for mobility and communication. The burden is thus removed from the health facility, and placed upon the family/household. Indeed, the case of Naphiri illustrates that access to health care in this context is dependent on informal caregivers, and as such access will be jeopardized for those who do not have access to caregivers. There is, to our knowledge, no empirical evidence to support such an assumption, as the available evidence from this study and from similar studies indicates that most people do in fact have access to informal caregiving, either at family or community level [9, 10, 54, 55]. An issue highlighted in previous research is that expression of symptoms and needs may be compromised by the presence of a guardian, or using guardians as proxies for patients [71]. Furthermore, the presence of guardians in treatment and care may compromise the patient-provider confidentiality, or add to patients’ feelings of being a burden to their surroundings, as expressed by Naphiri.

Similar to what has been extensively documented in previous studies [56–58, 60], this study found that both HIV and disability have financial implications for an individual and their immediate and extended household. Illness and disability often result in reduced ability to engage in income-generating activities, both for the individual with disability and the care-giver, thereby negatively affecting the livelihood of the family. The dependency of many people with disabilities on other family members means that health-seeking costs are often doubled, because someone has to accompany the disabled individual. Such costs could be transport costs, food costs and lack of income. Routines at health service level, such as calling people back for continued treatment on the following day, rather than completing all treatment procedures on the same day, add to the financial and practical burdens of patients and their households. As exemplified in the case of Naphiri, such financial implications reach much further than an individual or a household, and place an added financial burden on other family members outside of the immediate household.

## Conclusion

This research is exploratory research in progress, and as such more research is needed to draw generalizable conclusions. However, the case of Naphiri raises issues that are relevant for the broader context. While previous research has documented the challenges that people with disabilities face in accessing health services, the case of Naphiri highlights the complexities of access. There is a need for a more family and community oriented perspective in the organisation of health services, which implies support not just for the immediate patient, but also for their broader care system. Furthermore, access to health care is about more than just access to the actual health facility and treatment. Access is also about recognising barriers individuals and families face in attempting to seek health care, and the basis on which people make health care choices. The barriers extend far beyond getting to and from the service and accessing appropriate treatment. Barriers can be about the ability to make the choice and the necessary arrangements to seek treatment. As illustrated in the case of Naphiri this choice and these arrangements are not just dependent on Naphiri herself, but on her immediate and extended household.

Support can be divided into several categories, such as financial, practical and emotional support directed at the individual and his/her immediate and extended household. Support can also include more health services being provided at community and even household level. The life of an individual and the life of a household are holistic and interconnected, and cannot be divided into categories and compartments, and this has to be reflected in structures of care and support, which should have contextual, cultural and individual realities at the core.

In order to ensure equitable access to health care for all, policies, plans, programmes and research should apply a broader and more holistic focus, including family and community perspectives. Disability and HIV do not only affect the individual, but the whole household, and potentially the wider community and network of family, friends and neighbours. It is crucial to consider the interconnectedness of the challenges faced by an individual, a family and a household. Issues of health (physical and mental), disability, employment, education, infrastructure (transport/terrain) and poverty are all related and interconnected, and should be addressed as a whole in order to secure equity in health.

### Availability of data and materials

The raw data (case study and interviews with health providers) supporting the conclusions of this article are not freely available. This is to protect the anonymity of the informants. The depth of the information in the interview transcripts makes it possible to identify many of the informants based on their stories. Thus, the results presented in this article have been anonymised and altered slightly to ensure anonymity of the participants.

### Ethics and consent

The study was approved by the National Health Sciences Research Committee (NHSRC) of the Ministry of Health in Malawi (Reference number “Protocol #: 1051”). Informed consent to participate in the study was obtained from all participants.

### Consent for publication

n/a

## References

[1] United Nations (1948). The universal declaration of human rights.

[2] United Nations (2006). Convention on the rights of persons with disabilities.

[3] World Health Organization (WHO) (1978). Declaration of Alma Ata. International conference on primary health care. Alma Ata.

[4] London L (2007). Issues of equity are also issues of rights: Lessons from experiences in South Africa. BMC Public Health.

[5] United Nations (2011). Disability and the millennium development goals. A review of the MDG process and strategies for inclusion of disability issues in millennium development goal efforts.

[6] MacLachlan M, Swartz L (2009). Disability and international development: Towards inclusive global health.

[7] Eide AH, Loeb ME, Nhiwatiwa S, Munthali A, Ngulube TJ, van Rooy G, Eide AH, Ingstad B (2011). Living conditions among people with disabilities in developing countries. Disability and poverty: A global challenge.

[8] Evans R, Thomas F (2009). Emotional interactions and an ethics of care: Caring relations in families affected by HIV and AIDS. Emot Space Soc.

[9] Breen A, Swartz L, Flisher AJ, Joska JA, Corrigall J, Plaatjies L, McDonald DA (2007). Experience of mental disorder in the context of basic service reforms: The impact on caregiving environments in South Africa. Int J Environ Health Res.

[10] Levin K (2006). ‘I am what I am because of who we all are’: International perspectives on rehabilitation: South Africa. Pediatr Rehabil.

[11] Sandy PT, Kgole JC, Mavundla TR (2013). Support needs of caregivers: Case studies in South Africa. Int Nurs Rev.

[12] Eide AH, Mannan H, Khogali M, van Rooy G, Swartz L, Munthali A, Hem K-G, MacLachlan M, Dyrstad K (2015). Perceived barriers for accessing health services among individuals with disability in four African countries. PLoS One.

[13] Maclachlan M, Mannan H, McAuliffe E (2011). Access to health care of persons with disabilities as an indicator of equity in health systems. Open Med.

[14] World Health Organization and The World Bank (2011). World report on disability.

[15] Elwan A (1999). Poverty and disability: A survey of the literature.

[16] Groce N, Trani JF (2009). Millennium development goals and people with disabilities. Lancet.

[17] Eide AH, Ingstad B (2011). Disability and poverty: A global challenge.

[18] Braathen SH, Vergunst R, Mji G, Mannan H, Swartz L (2013). Understanding the local context for the application of global mental health: A rural South African experience. Int Health.

[19] Keikelame MJ, Swartz L (2013). A lay carer’s story about epilepsy in an urban South African context: They call it an illness of falling or an illness of fitting because a person shakes and eventually falls. Epilepsy Behav.

[20] Garland-Thomson R (2005). Feminist disability studies. Signs.

[21] Good BJ, Good MDV (1994). In the subjunctive mode: Epilepsy narratives in Turkey. Soc Sci Med.

[22] Leonardi M, Bickenbach J, Ustun TB, Kostanjsek N, Chatterji S (2006). The definition of disability: What is in a name?. Lancet.

[23] World Health Organization (2001). International Classification of Functioning, Disability and Health (ICF).

[24] Shakespeare T (2014). Disability rights and wrongs revisited.

[25] Disability: Beyond the medical model. Lancet. 2009;374(9704):1793. 10.1016/S0140-6736(09)62043-2 19944842

[26] Shakespeare T, Iezzoni LI, Groce NE (2009). Disability and the training of health professionals. Lancet.

[27] Ingstad B, Munthali AC, Braathen SH, Grut L (2012). The evil circle of poverty: A qualitative study of malaria and disability. Malar J.

[28] Officer A, Groce NE (2009). Key concepts in disability. Lancet.

[29] Grut L, Mji G, Braathen SH, Ingstad B (2012). Accessing community health services: Challenges faced by poor people with disabilities in a rural community in South Africa. AJOD.

[30] Hwang K, Johnston M, Tulsky D, Wood K, Dyson-Hudson T, Komaroff E (2009). Access and coordination of health care service for people with disabilities. JDPS.

[31] Stein MA, Stein PJ, Weiss D, Lang R (2009). Health care and the UN Disability Rights Convention. Lancet.

[32] Grut L, Sanudi L, Braathen SH, Jürgens T, Eide AH (2015). Access to tuberculosis services for individuals with disability in rural Malawi, a qualitative study. PLoS One.

[33] United Nations (2015). Human Development Index. United Nations Development Programme.

[34] Loeb M, Eide AH (2004). Living conditions among people with activity limitations in Malawi.

[35] Evans R, Atim A (2011). Care, disability and HIV in Africa: diverging or interconnected concepts and practices?. TWQ.

[36] Lane T, Cluver L, Operario D (2015). Young carers in South Africa: Tasks undertaken by children in households affected by HIV infection and other illness. Vulnerable Child Youth Stud.

[37] Wang Q, Brenner S, Leppert G, Banda TH, Kalmus O, De Allegri M (2015). Health seeking behaviour and the related household out-of-pocket expenditure for chronic non-communicable diseases in rural Malawi. Health Policy Plann.

[38] Loevinsohn M (2015). The 2001-03 famine and the dynamics of HIV in Malawi: A natural experiment. PLoS One.

[39] Laar A, Fiaveh D, Laar M, Boatemaa S, Abugri J, El-Adas A, Amenyah R, Atuahene K, Adjei AA, Quakyi I (2014). Profiles of HIV-affected households in Ghana. Health.

[40] Groce NE (2003). HIV/AIDS and people with disability. Lancet.

[41] Groce NE (2005). HIV/AIDS and individuals with disability. Health Hum Rights.

[42] Groce NE, Rohleder P, Eide AH, MacLachlan M, Mall S, Swartz L (2013). HIV issues and people with disabilities: a review and agenda for research. Soc Sci Med.

[43] Rohleder P, Braathen SH, Swartz L, Eide AH (2009). HIV/AIDS and disability in Southern Africa: a review of relevant literature. Disabil Rehabil.

[44] Rohleder P, Swartz L, Aggleton P, Boyce P, Moore HL, Parker R (2012). Disability, sexuality and sexual health. Understanding global sexualities: New frontiers.

[45] Hanass-Hancock J (2009). Disability and HIV/AIDS - a systematic review of literature on Africa. J Int AIDS Soc.

[46] Hanass-Hancock J, Nixon SA (2009). The fields of HIV and disability: past, present and future. J Int AIDS Soc.

[47] Sander-Staudt M (2014). Care ethics.

[48] Tronto J (1993). Moral boundaries: A political argument for an ethic of care.

[49] Fisher B, Tronto J, Abel E, Nelson M (1990). Toward a feminist theory of caring. Circles of care.

[50] Engster D (2005). Rethinking care theory: The practice of caring and the obligation to care. Hypatia.

[51] Kittay EF, Jennings B, Wasunna AA (2005). Dependency, difference and the global ethic of longterm care. J Polit Philos.

[52] Tronto J, Holstein MB, Mitzen PB (2001). An ethic of care. Ethics in community-based elder care.

[53] Kaseje DOC, Mpenda B, WHO (2002). Appendix D: The African perspective. Ethical choices in long-term care: What does justice require?.

[54] Viana MC, Gruber MJ, Shahly V, Alhamzawi A, Alonso J, Andrade LH (2013). Family burden related to mental and physical disorders in the world: results from the WHO World Mental Health (WMH) surveys. Rev Bras Psiquiatr.

[55] Colvin CJ, Smith HJ, Swartz A, Ahs JW, de Heer J, Opiyo N (2013). Understanding careseeking for child illness in sub-Saharan Africa: a systematic review and conceptual framework based on qualitative research of household recognition and response to child diarrhoea, pneumonia and malaria. Soc Sci Med.

[56] Goudge J, Gilson L, Russell S, Gumede T, Mills A (2009). Affordability, availability and acceptability barriers to health care for the chronically ill: longitudinal case studies from South Africa. BMC Health Serv Res.

[57] Goudge J, Gilson L, Russell S, Gumede T, Mills A (2009). The household costs of health care in rural South Africa with free public primary care and hospital exemptions for the poor. Trop Med Int Health.

[58] Goudge J, Russel S, Gilson L, Gujmede T, Tollman S, Mills A (2009). Illness-related impoverishment in rural South Africa: Why does social protection work for some households but not others?. J Int Dev.

[59] Goudge J, Russell S, Gilson L, Molyneux C, Hanson K (2009). Household experiences of ill-health and risk protection mechanisms. J Int Dev.

[60] Naidu V, Harris G (2005). The impact of HIV/AIDS morbidity and mortality on households – A review of household studies. S Afr J Econ.

[61] Miles M (2002). Children with Hydrocephalus and Spina Bifida in East Africa: Can family and community resources improve the odds?. Disabil Soc.

[62] Yin RK (2013). Case study research: Design and methods.

[63] Stake RE (2006). Multiple case study analysis.

[64] Crowe S, Cresswell K, Robertson A, Huby G, Avery A, Sheikh A (2011). The case study approach. BMC Med Res Methodol.

[65] Read S, Maslin-Prothero S (2011). The involvement of users and carers in health and social research: the realities of inclusion and engagement. Qual Health Res.

[66] Capri C, Coetzee O (2012). On the unethicality of disablism: Excluding intellectually impaired individuals from participating in research can be unethical. AJOD.

[67] McDonald KE, Kidney CA (2012). What is right? Ethics in intellectual disabilities research. JPPID.

[68] McDonald KE, Kidney CA, Patka M (2013). ‘You need to let your voice be heard’: research participants’ views on research. J Intellect Disabil Res.

[69] Malawi Ministry of Health (2011). Malawi health sector strategic plan 2011-2016.

[70] Stephens R (2013). Machinga district hospital: Medical drugs give hope.

[71] Kritzinger J, Schneider M, Swartz L, Braathen SH (2014). “I just answer ‘yes’ to everything they say”: access to health care for deaf people in Worcester, South Africa and the politics of exclusion. Patient Educ Couns.

